# Charge Transport in the Presence of Correlations and Disorder: Organic Conductors and Manganites

**DOI:** 10.3390/ma17071524

**Published:** 2024-03-27

**Authors:** Emil Tafra, Mario Basletić, Tomislav Ivek, Marko Kuveždić, Nikolina Novosel, Silvia Tomić, Bojana Korin-Hamzić, Matija Čulo

**Affiliations:** 1 Department of Physics, Faculty of Science, University of Zagreb, Bijenička Cesta 32, HR-10000 Zagreb, Croatia; etafra@phy.hr (E.T.); basletic@phy.hr (M.B.);; 2 Institut za Fiziku, Bijenička Cesta 46, HR-10000 Zagreb, Croatia; tivek@ifs.hr (T.I.); nnovosel@ifs.hr (N.N.); stomic@ifs.hr (S.T.); hamzic.bojana@gmail.com (B.K.-H.)

**Keywords:** electron localization, disorder effects, correlation effects, organic conductors, manganites, Mott–Anderson localization

## Abstract

One of the most fascinating aspects of condensed matter is its ability to conduct electricity, which is particularly pronounced in conventional metals such as copper or silver. Such behavior stems from a strong tendency of valence electrons to delocalize in a periodic potential created by ions in the crystal lattice of a given material. In many advanced materials, however, this basic delocalization process of the valence electrons competes with various processes that tend to localize these very same valence electrons, thus driving the insulating behavior. The two such most important processes are the Mott localization, driven by strong correlation effects among the valence electrons, and the Anderson localization, driven by the interaction of the valence electrons with a strong disorder potential. These two localization processes are almost exclusively considered separately from both an experimental and a theoretical standpoint. Here, we offer an overview of our long-standing research on selected organic conductors and manganites, that clearly show the presence of both these localization processes. We discuss these results within existing theories of Mott–Anderson localization and argue that such behavior could be a common feature of many advanced materials.

## 1. Introduction

The basic classification of materials in condensed matter physics is based on their electrical conductivity, a quantity that describes how well they can conduct electricity. Alternatively, the concept of resistivity can be used which is the reciprocal of electrical conductivity. According to this basic classification, the materials are usually divided into three groups: metals, semiconductors, and insulators. Between a good metal and a good insulator, electrical resistivity ρ can vary by more than twenty orders of magnitude, which is a truly amazing difference. Such a difference can be explained only within the quantum mechanical description of a solid crystalline material, which is the famous band theory developed during the late 1920s by Bethe [1], Sommerfeld [2], and Bloch [3].

According to the band theory, eigenstates of a periodic crystal potential are completely delocalized and are described by Bloch wave functions, while eigenenergies can take only values in a certain range, which results with an electronic structure consisting of energy bands separated by energy gaps. The highest/top band of the set of completely full energy bands is known as the valence band. The next higher-energy band is named the conduction band, which can be completely empty or partially filled. In case of metals, the valence band is completely full and the conduction band is partially filled, or the valence and conduction bands overlap, so that there is a finite density of states at the Fermi level, $E_{F}$. On the other hand, in case of semiconductors and insulators, the valence band is completely filled with valence electrons and is separated by an energy gap from the conduction band, so that the density of states at $E_{F}$ is zero. The only difference between the semiconductors and insulators is in the size of the gap, which in the case of insulators is so large that their electrical conductivity is negligible.

The profile of density of states around $E_{F}$ has a large impact not only on the value of resistivity, but also on its temperature (*T*) dependence. The resistivity of metals usually has low values and increases with increasing temperature (dρ/dT>0) in a power-law manner, predominantly due to a *T*-variation of the scattering time, since the number of electrons is almost constant in *T*. On the other hand, the resistivity of semiconductors usually has significantly higher values and decreases with increasing temperature (dρ/dT<0) with predominantly exponential behavior ρ(T)∝exp(Δ/T) due to the increasing number of electrons that are activated across energy gap 2Δ. Since there is no qualitative difference between semiconductors and insulators, the *T* dependence of resistivity with dρ/dT<0 is in the literature referred to as a semiconductor-like or an insulator-like behavior interchangeably. Here, we refer to such *T* dependence as insulator-like behavior.

Many advanced materials, however, cannot be captured by such a simple picture based on the conventional band theory. Usually, the band theory for these materials predicts a partially filled band at $E_{F}$, i.e., the metallic state, while the experiments indicate quite the opposite, insulator-like behavior. There are two main mechanisms that can result in an insulating behavior from partially filled bands. One is driven by the localization of conduction electrons induced by correlation effects, and the other by the localization induced by disorder effects. The first localization mechanism, based on the correlation effects, is typical for the so-called strongly correlated systems, the most important examples of which are transition metal oxides and organic conductors. In contrast to conventional materials, here, the conduction electrons interact strongly with one another, and therefore they cannot move freely through the crystal. One of the simplest and most common theoretical approaches for these strongly correlated systems is the model based on the Hubbard Hamiltonian with a half-filled band, i.e., with one electron per crystal site [4]. Besides the interaction of conduction electrons with the periodic crystal potential, the Hubbard Hamiltonian also takes into account the effective Coulomb repulsion between two electrons on the same crystal site, the so-called Hubbard term *U*.

A useful concept in the Hubbard model is the correlation strength U/W, where *W* is the bandwidth of the half-filled band. If the correlation strength is small, U/W<<1, the electrons can easily delocalize so that the Hubbard model can still predict a metallic state with a half-filled band, in line with the predictions of the conventional band theory. Conversely, if the correlation strength is large, U/W>>1, the electrons try to avoid being on the same crystal site and remain localized in such a way that there is one electron per crystal site. In this case, the Hubbard model predicts an insulating state, in contrast with the conventional band theory. As such, the so-called Mott localization of conduction electrons is accompanied by a splitting of the half-filled band into an upper Hubbard band which is completely empty and a lower Hubbard band which is completely filled. In other words, the Mott insulating state is accompanied by opening of the energy gap of the order *U* at $E_{F}$, which is usually referred to as the Mott–Hubbard gap [4].

Another common model for the strongly correlated systems is the extended Hubbard model with a quarter-filled band, i.e., one electron per two crystal sites [5,6]. The extended Hubbard model, besides the interaction with the periodic crystal potential and the Hubbard term *U*, also takes into account the so-called extended Hubbard term *V* that describes the effective Coulomb repulsion between two electrons on neighboring crystal sites. Here, the correlation strength is defined as V/W, where *W* is the bandwidth of the quarter-filled band. For small V/W<<1, the extended Hubbard model produces the same prediction as the conventional band theory, namely a metallic state with a quarter-filled band. On the other hand, for large V/W>>1, the extended Hubbard model, in contrast to the band theory, predicts an insulating state due to the localization of conduction electrons caused by strong inter-site repulsion *V*. In this case, the conduction electrons try to avoid occupying neighboring crystal sites and therefore localize on every other crystal site, thus forming a long-range charge-ordered (CO) state. Localization of electrons due to CO is accompanied by a splitting of the quarter-filled band into an upper band which is completely empty and a lower band which is completely filled, i.e., by opening of the energy gap of the order of *V* at $E_{F}$, which is usually referred to as the CO gap [7].

The second mechanism of localization of conduction electrons, based on the disorder effects, is typical for disordered, non-crystalline systems, the most important examples of which are organic polymers and amorphous alloys. In contrast to conventional materials with periodic crystal potential, due to the disorder potential, the eigenstates are not described by the Bloch wave functions and eigenenergies do not involve well-defined energy bands and energy gaps. Although the density of states at $E_{F}$ is finite, a material does not exhibit the metallic behavior as states at $E_{F}$ are localized and conduction electrons cannot move freely through the solid.

The most common theoretical treatment of a disordered potential is the Anderson model, which assumes a spatially periodic arrangement of potential wells but with randomly distributed depths [8,9]. A useful concept in the Anderson model is disorder strength δ, which measures the degree to which the potential well depths are scattered around the average value. If the disorder strength is small, δ<<1, the electrons can easily delocalize, and the Anderson model predicts a metallic state with a partially filled band, in line with the predictions of the conventional band theory. On the other hand, if the disorder strength is large, δ>>1, a significant energy difference between neighboring sites leads to vanishingly small mixing of their electron wave functions, which prevents propagation and in turn leads to localization of the conduction electrons inside the disordered medium. In this case, the Anderson model predicts an insulating state, in contrast with the conventional band theory. As such, the so-called Anderson localization of conduction electrons does not result in an opening of an energy gap at $E_{F}$, but with a change in the character of states at $E_{F}$ from delocalized to localized [8,9,10,11].

In contrast to the correlation-induced (Mott or CO) localization of conduction electrons, which drives the insulating behavior by opening of an energy gap at $E_{F}$, the disorder-induced (Anderson) localization of electrons drives the insulating behavior by changing the character of states at $E_{F}$ from delocalized to localized. As a consequence, charge transport in the Mott and CO insulators takes place by the activation of electrons across correlation gap 2Δ, so that the resistivity is expected to follow a simple activated behavior ρ(T)∝exp(Δ/T). Examples of such activated behavior have been recently detected in organic conductors β′-EtMe_3_Sb[Pd(dmit)_2_]_2_ [12] and δ′-(BEDT-TTF)_2_CF_3_CF_2_SO_3_ [13], as well as in rare-earth titanates (Y,La)TiO_3_ and (Y,Ca)TiO_3_ [14]. However, charge transport in the Anderson insulators is observed to take place via hopping of conduction electrons among localized states in the vicinity of $E_{F}$. The hopping at low *T* usually occurs between distant localized states, so that the resistivity follows the variable range hopping (VRH) mechanism, $\rho \left(T\right)\propto \exp{\left(T_{0}/T\right)}^{1/(d+1)}$. Here, $T_{0}\propto 1/\left(n_{F}\xi^{d}\right)$ is the characteristic Mott temperature, $n_{F}$ is the density of localized states at $E_{F}$, ξ is the localization length, and *d* is the dimensionality of the system [10]. Typical examples of such hopping conductivity have been detected in amorphous semiconductors such as Si and Ge [15] and recently in the nanocrystalline carbon thin films [16], *n*-type ultra-nanocrystalline diamond [17] and lower-rim-substituted calixarene derivatives in thin films [18]. With increasing temperature, the hopping among localized states of variable range usually crosses over to nearest-neighbor hopping (NNH), which has the same *T* dependence as the simple activated behavior, $\rho \left(T\right)\propto \exp\left(\Delta_{\mathrm{NNH}}/T\right)$ [19]. Here, it is important not to confuse $\Delta_{\mathrm{NNH}}$ with Δ, since the latter is related to a real gap in the density of states, as in Mott and CO insulators, while the former simply represents the activation energy for a hopping process in Anderson insulators that do not have a gap in the density of states.

Here, we present an overview of our long-standing study on selected organic conductors and manganites that clearly show how neither the Mott/CO localization nor the Anderson localization alone can fully capture their insulating behavior. By doing so, we do not wish to focus on all the aspects of a specific material, but only on those results that are crucial for understanding the nature of their insulating behavior. First, we note that conventional band theory predicts a metallic state, while advanced theoretical considerations imply the correlation-induced (Mott or CO) insulating phase. Our resistivity measurements confirm insulator-like behavior, but instead of the expected simple activation, we find clear fingerprints of VRH and/or NNH behavior, indicating the disorder-induced (Anderson) insulating phase. Surprisingly, instead of strengthening the insulating behavior, an increase in disorder counter-intuitively pushes the system towards the metallic state, in sharp contrast to the Anderson scenario. In this regard, we reveal a clear pattern of resistivity behavior that is observed in all these materials and can be understood only within more complex theories of Mott–Anderson localization that study the electron localization in the presence of both, correlations and disorder. We discuss this theory in more detail after the experimental overview of selected compounds. We suggest that this finding could be a generic feature of strongly correlated systems.

## 2. κ-(BEDT-TTF)_2_Cu_2_(CN)_3_

**Figure 1:**
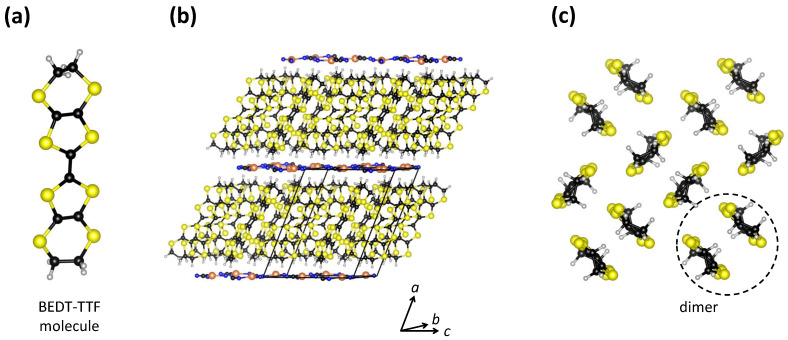
(**a**) Schematic drawing of a BEDT-TTF molecule. (**b**) Layered crystal structure of κ-(BEDT-TTF)${\;\;}_{2}$Cu${\;\;}_{2}$(CN)${\;\;}_{3}$. The unit cell is indicated by black lines. (**c**) View of a BEDT-TTF layer in the bc plane projected along the *a* axis. Sulphur, carbon, copper, nitrogen, and hydrogen atoms are shown in yellow, black, orange, blue, and gray, respectively. This figure is based on data from Ref. [29].

As a first example of a material in which the charge transport is strongly influenced by both correlations and disorder, let us consider organic conductor κ-(BEDT-TTF)_2_Cu_2_(CN)_3_, or κ-Cu for short. Here, BEDT-TTF stands for organic molecule bis(ethyle-nedithio)tetrathiafulvalene (Figure 1a). This material has attracted a lot of attention because it was proposed to be one of the best candidates for a quantum spin liquid with itinerant spinons [20], theoretically presented by Anderson more than 50 years ago [21]. In addition, it also possesses a rich phase diagram under pressure, featuring a Mott metal–insulator (MI) transition, unconventional superconductivity, and non-Fermi-liquid behavior [22].

κ-Cu has a layered crystal structure in which organic BEDT-TTF (cation) layers and inorganic Cu_2_(CN)_3_ (anion) layers are alternately stacked one on another [23,24] (Figure 1b). The BEDT-TTF molecules within the organic layers are paired in dimers that form a triangular lattice with each dimer oriented approximately perpendicular to its neighbors, which is the so-called κ-type arrangement (Figure 1c)). Semiempiricial and first principle band structure calculations [25,26,27] show the cation-derived character of κ-(BEDT-TTF)_2_Cu_2_(CN)_3_ band structure around the Fermi level, whereas copper occurs in the Cu^1+^ oxidation state and has a filled 3*d* shell. In other words, electrical conduction occurs only in the organic BEDT-TTF layers. These calculations also show that each BEDT-TTF dimer carries one hole, which implies that the valence band is half-filled and that therefore the system is expected to exhibit metallic behavior. Transport experiments [28], however, indicate that κ-Cu is an insulator, which is usually ascribed to the Mott localization of conducting charge carriers within a half-filled band.

Consequently, the majority of theoretical models are based on a half-filled Hubbard Hamiltonian on an anisotropic triangular lattice [30]. As mentioned in Section 1, a useful concept in the Hubbard model is the correlation strength, U/W, where in this case bandwidth *W* corresponds to the bands created by BEDT-TTF dimers and Hubbard term *U* corresponds to the effective Coulomb repulsion between two electrons on the same BEDT-TTF dimer. Correlation strength U/W in κ-Cu has an intermediate value of U/W≈1 [24,26,31,32], which implies that it lies close to the MI transition. Indeed, a hydrostatic pressure of only about 4 kbar increases *W* sufficiently to destroy the insulating state and establish the metallic or even the superconducting state [28].

In the literature, therefore, κ-Cu at ambient pressure is usually considered to be a Mott insulator, which implies the existence of a correlation-induced Mott–Hubbard gap $2\Delta_{\mathrm{Mott}}$ at $E_{F}$. However, the two most powerful probes for the detection of an energy gap, resistivity and infrared spectroscopy, cast doubts that κ-Cu is a simple Mott insulator. In contrast to expectations, no clear cut-off frequency was observed in the infrared spectra [33,34] and no simple activated behavior $\rho \left(T\right)\propto \exp\left(\Delta_{\mathrm{Mott}}/T\right)$ was found in the *T* dependence of the resistivity [35]. Our detailed analysis of resistivity [35], in fact, showed that charge transport in κ-Cu at low *T* takes place via the VRH mechanism $\rho \left(T\right)\propto \exp{\left(T_{0}/T\right)}^{1/(d+1)}$. The best fit was obtained for VRH in two dimensions (d=2), in accordance with the quasi-2D nature of κ-Cu. The 2D VRH mechanism was additionally confirmed by magnetotransport measurements [36].

**Figure 2:**
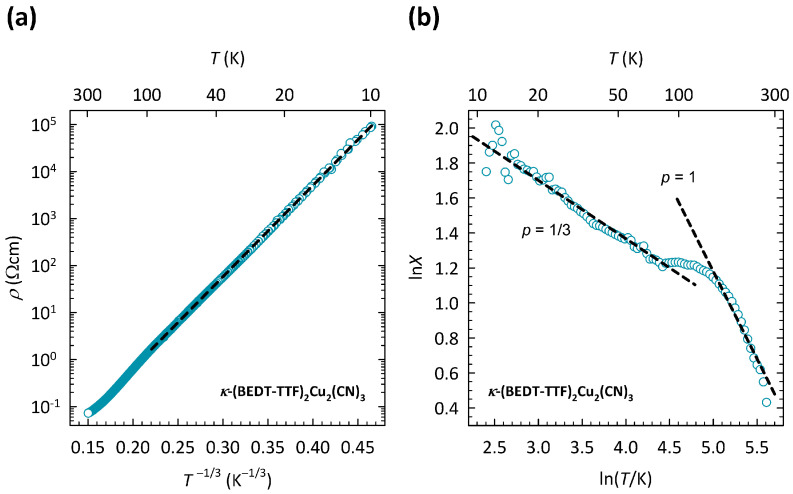
(**a**) Temperature dependence of resistivity for κ-(BEDT-TTF)${\;\;}_{2}$Cu${\;\;}_{2}$(CN)${\;\;}_{3}$ on a logρ-$T^{-1/3}$ plot. Black dashed line is a fit to the 2D VRH mechanism $\rho \left(T\right)\propto \exp{\left(T_{0}/T\right)}^{1/3}$. (**b**) Temperature dependence of the special logarithmic resistivity derivative $X=-d\ln\rho /\left(d\ln T\right)=p{\left(C/T\right)}^{p}$ on a double logarithmic plot. Black dashed lines correspond to slopes p=1/3 for 2D VRH and p=1 for NNH, where *p* is the exponent in a general expression for resistivity $\rho \left(T\right)\propto \exp{\left(C/T\right)}^{p}$ (see text). The figure is based on data from Ref. [37].

The presence of the 2D VRH mechanism for κ-Cu proposed in our transport study [35] is depicted here in Figure 2a. The data are shown on a logρ-$T^{-1/3}$ plot, suitable for 2D VRH. As can be clearly seen, the ρ(T) data in the *T*-range 10–100 K can be fitted to the 2D VRH mechanism almost perfectly, which is indicated by a black dashed line. Note that the fit to 2D VRH covers the change in resistivity by almost five orders of magnitude. The deviation from 2D VRH above 100 K was attributed to the crossover to the NNH regime [35].

As we showed in Ref. [37], the crossover from 2D VRH to NNH can be most clearly seen in the behavior of special logarithmic resistivity derivative X=−dlnρ/(dlnT), introduced in several papers [38,39,40,41,42]. In this case, one starts from a more general expression for resistivity $\rho \left(T\right)\propto \exp{\left(C/T\right)}^{p}$ that is common to both VRH ($C=T_{0}$ and p=1/(d+1)) and NNH ($C=\Delta_{\mathrm{NNH}}$ and p=1). By taking the special derivative, one obtains $X=-d\ln\rho /\left(d\ln T\right)=p{\left(C/T\right)}^{p}$, which enables a direct determination of exponent *p* from the slope in the plot of ln*X* vs. ln*T*.

Such a ln*X*-ln*T* plot for κ-Cu is shown in Figure 2b. As we can see, in the *T*-range of 10–100 K, the slope p=1/3, which corresponds to the 2D VRH mechanism, in agreement with the fitting procedure outlined in Figure 2a. The change in slope around 100 K is most naturally ascribed to the crossover from 2D VRH to NNH, which is a typical behavior of hopping conductivity and is always expected to occur with increasing temperature. Indeed, at high *T*, the slope p=1, which corresponds to the *T* dependence of resistivity $\rho \left(T\right)\propto \exp\left(\Delta_{\mathrm{NNH}}/T\right)$, as expected in the NNH regime.

The presence of hopping conductivity in κ-Cu implies a significant level of disorder within the conducting BEDT-TTF layers, which is somewhat surprising, since the corresponding single crystals are nominally clean. The presence of disorder within the conducting layers was also hinted at by a relaxor-like behavior in the dielectric response [35,43]. As we argued in detail in Ref. [35], the source of the disorder is to be found within the insulating Cu_2_(CN)${\;\;}_{3}$ layers, more precisely in those CN groups that lie on inversion centers and are therefore crystallographically disordered. We proposed that this disorder within the insulating layers is effectively transferred to the conducting layers via strong hydrogen bonds between the ethylene groups of the BEDT-TTF molecules and disordered CN groups.

The presence of hopping conductivity in κ-Cu further implies the existence of disorder-induced localized states at $E_{F}$. This means that, in contrast to common beliefs, the insulating behavior of κ-Cu is to be understood within the theory of Anderson localization of conducting charge carriers, rather than within the theory of Mott localization. In other words, κ-Cu seems to be closer to Anderson-type than to Mott-type insulators. In contrast to Mott insulators, which have a clear gap at $E_{F}$, Anderson insulators are gapless, with the states at $E_{F}$ that are localized by a strong disorder potential [44]. The stronger the disorder potential, the more localized the states at $E_{F}$ and consequently the more insulating the system. In order to study such disorder-induced localization, one would have to control the level of disorder in single crystals of κ-Cu, which is experimentally very demanding. One way of achieving it is by high-intensity X-ray irradiation, which introduces defects in the crystal structure and therefore increases the level of structural disorder. Such an experiment was conducted on κ-Cu and indeed, a significant impact of irradiation on resistivity was observed [45]. This change in resistivity, however, is very hard to directly correlate with the increase in the structural disorder, since X-ray irradiation simultaneously also changes the chemical potential by doping the system with free charge carriers.

In order to avoid such problems, our group rather focused on the chemical substitution in κ-Cu, where the Cu${\;\;}_{2}$(CN)${\;\;}_{3}$ anion is replaced by Ag${\;\;}_{2}$(CN)${\;\;}_{3}$ or B(CN)${\;\;}_{4}$ anions [37]. The most relevant consequence of such substitution is a large change in the level of structural disorder within the conducting BEDT-TTF layers, without a simultaneous shift in the chemical potential. Namely, κ-(BEDT-TTF)${\;\;}_{2}$Ag${\;\;}_{2}$(CN)${\;\;}_{3}$, or shorter κ-Ag, similarly to κ-Cu, also hosts the disorder within the insulating Ag${\;\;}_{2}$(CN)_3_ layers due to CN groups that reside on inversion centers, and this disorder is transferred to the conducting BEDT-TTF layers via hydrogen bonds. Due to longer contacts between the disordered CN groups and terminal ethylene groups of BEDT-TTF molecules, however, the degree of disorder within the conducting BEDT-TTF layers in κ-Ag is significantly lower than in κ-Cu [31,46]. On the other hand, κ-(BEDT-TTF)${\;\;}_{2}$B(CN)${\;\;}_{4}$, or shorter κ-B, contains no crystallographically disordered CN groups, since the B(CN)${\;\;}_{4}$ anion is orientationally ordered already at room temperature [47]. This means that κ-B has the lowest level, κ-Ag has an intermediate level, and κ-Cu has the highest level of structural disorder.

**Figure 3:**
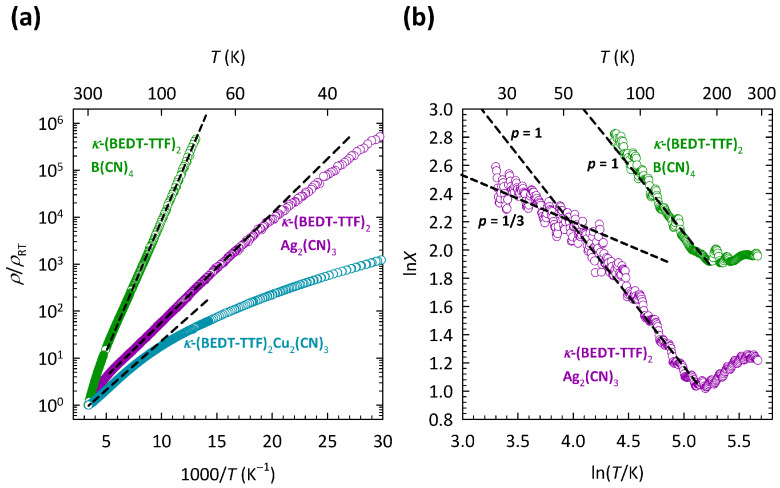
(**a**) Temperature dependence of resistivity normalized to room temperature $\rho /\rho_{\mathrm{RT}}$ for κ-(BEDT-TTF)${\;\;}_{2}$Cu${\;\;}_{2}$(CN)${\;\;}_{3}$ (dark cyan symbols), κ-(BEDT-TTF)${\;\;}_{2}$Ag${\;\;}_{2}$(CN)${\;\;}_{3}$ (violet symbols), and κ-(BEDT-TTF)${\;\;}_{2}$B(CN)${\;\;}_{4}$ (green symbols) on a logρ - 1/T plot. The data for κ-(BEDT-TTF)${\;\;}_{2}$Cu${\;\;}_{2}$(CN)${\;\;}_{3}$ are the same as in Figure 2a. Black dashed lines are fits to the NNH mechanism $\rho \left(T\right)\propto \exp\left(\Delta_{\mathrm{NNH}}/T\right)$. (**b**) Temperature dependence of special logarithmic resistivity derivative $X=-d\ln\rho /\left(d\ln T\right)=p{\left(C/T\right)}^{p}$ on a double logarithmic plot for κ-(BEDT-TTF)${\;\;}_{2}$Ag${\;\;}_{2}$(CN)${\;\;}_{3}$ (violet symbols) and κ-(BEDT-TTF)${\;\;}_{2}$B(CN)${\;\;}_{4}$ (green symbols). Black dashed lines correspond to slopes p=1/3 for 2D VRH and p=1 for NNH, where *p* is the exponent in a general expression for resistivity $\rho \left(T\right)\propto \exp{\left(C/T\right)}^{p}$ (see text). The figure is based on data from Ref. [37].

The comparison of transport properties between the three compounds, κ-Cu, κ-Ag, and κ-B outlined in our recent study [37], turned out to be very instructive. This comparison is presented here in Figure 3a, which shows the *T* dependence of the resistivity for all three compounds. The resistivities are normalized to room temperature values $\rho_{\mathrm{RT}}$ in order to highlight the difference in *T* dependence between the compounds with different anions. The values of $\rho_{\mathrm{RT}}$ are approximately 70 mΩcm, 450 Ωcm, and 260 Ωcm for κ-Cu, κ-Ag, and κ-B, respectively. The corresponding *T* dependence of special derivatives X=−dlnρ/(dlnT) for κ-Ag and κ-B is shown in Figure 3b. As we can see, with increasing *T*, the slope *p* in κ-Ag changes value from p=1/3 to p=1, which is very similar to the behavior of *p* in κ-Cu shown in Figure 2b. This change in slope *p* in κ-Ag was, as in κ-Cu, ascribed to the crossover from the 2D VRH to the NNH mechanism [37]. In the case of κ-B, due to very high resistances, ρ(T) data were recorded only down to 80 K, so that only the NNH regime with p=1 was observed.

As Figure 3a clearly illustrates, the most disordered compound, κ-Cu, exhibits the weakest insulating behavior, while the least disordered compound, κ-B, exhibits the strongest insulating behavior. In other words, a large increase in the structural disorder from κ-B, across κ-Ag towards κ-Cu, strongly increases the conductivity of the system, i.e., pushes the system towards the metallic state. Such behavior is, in fact, counter-intuitive and is in sharp contrast with the earlier proposed theory of Anderson localization.

The behavior of charge transport in κ-Cu and its sister compounds, therefore, cannot be fully captured either solely with the theory of Mott localization or solely with the theory of Anderson localization. As we argued in detail in Ref. [37], such behavior can be understood only within recent, more complex theories of Mott–Anderson localization [44,48,49]. The ways in which these theories can account for counter-intuitive disorder-induced increase in conductivity is discussed in Section 5.

## 3. α-(BEDT-TTF)${\;\;}_{2}$I${\;\;}_{3}$

As a second example of a material in which the charge transport is affected by correlations and disorder, let us consider organic conductor α-(BEDT-TTF)${\;\;}_{2}$I${\;\;}_{3}$. This compound is based on the same organic molecule bis(ethylenedithio)tetrathiafulvalene as the materials described in Section 2 and has been intensively studied due to a metal-to-insulator transition at 135 K into a charge-ordered ground state with CO-induced ferroelectricity [50,51,52,53,54]. It attracted a lot of attention also because of other intriguing phenomena such as a photo-induced phase transition [55,56], Dirac fermions under hydrostatic pressure [56,57,58], and unconventional superconductivity under uniaxial pressure [59].

**Figure 4:**
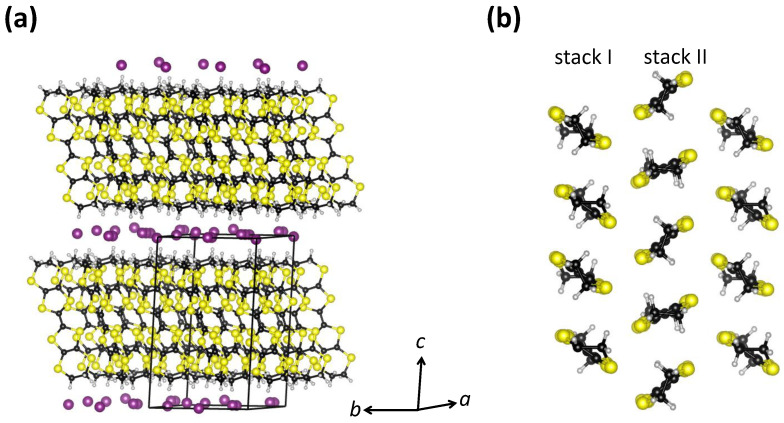
(**a**) Layered crystal structure of α-(BEDT-TTF)${\;\;}_{2}$I${\;\;}_{3}$. The unit cell is indicated by black lines. (**b**) View of a BEDT-TTF layer in the ab plane projected along the *c*-axis. Sulfur, carbon, iodine, and hydrogen atoms are shown in yellow, black, violet, and gray, respectively. This figure is based on data from Ref. [62].

α-(BEDT-TTF)${\;\;}_{2}$I${\;\;}_{3}$ has a layered crystal structure in which organic BEDT-TTF layers and inorganic I${\;\;}_{3}$ layers are alternately stacked one on another [60] (Figure 4a). The BEDT-TTF molecules within the organic layers in α-(BEDT-TTF)${\;\;}_{2}$I${\;\;}_{3}$ form a herringbone structure and are organized in a triangular lattice with two types of stacks, which is the so-called α-type arrangement. As in the case of κ-BEDT-TTF, the band structure calculations show that the band structure shape of the whole system around the Fermi level is predominantly determined by the molecular BEDT-TTF subsystem [61]. Therefore, the charge transport only takes place within the conducting BEDT-TTF layers, so that similar to κ-(BEDT-TTF)${\;\;}_{2}$Cu${\;\;}_{2}$(CN)${\;\;}_{3}$, α-(BEDT-TTF)${\;\;}_{2}$I${\;\;}_{3}$ also exhibits quasi-2D electronic properties [60] (Figure 4b). Finally, in α-(BEDT-TTF)${\;\;}_{2}$I${\;\;}_{3}$, the BEDT-TTF molecules are not paired in dimers, in contrast to the κ-type arrangement from Section 2. This means that according to stoichiometry, each BEDT-TTF molecule carries half a hole, i.e., the valence band is quarter-filled and therefore α-(BEDT-TTF)${\;\;}_{2}$I${\;\;}_{3}$ exhibits metallic conductivity. Indeed, at high temperatures, α-(BEDT-TTF)${\;\;}_{2}$I${\;\;}_{3}$ does exhibit metal-like behavior (dρ/dT>0), but below 135 K, there is a phase transition to an insulating CO ground state, which is confirmed by synchrotron X-ray diffraction [62], infrared spectroscopy [63,64,65,66], ${\;\;}^{13}$C NMR [67], transport measurements, and dielectric spectroscopy [66]. Here, it is worth mentioning that α-(BEDT-TTF)${\;\;}_{2}$I${\;\;}_{3}$ at high *T*, in fact, exhibits a semimetallic nature, with small electron and hole pockets at the Fermi level, as suggested by DFT calculations [68] and confirmed by our magnetotransport measurements [69]. This, however, does not affect the main conclusions we want to emphasize here.

A widely accepted mechanism for driving the insulating CO ground state of α-(BEDT-TTF)${\;\;}_{2}$I${\;\;}_{3}$ is based on a Hubbard Hamiltonian for a quarter-filled system that takes into account the Hubbard term *U* and the extended Hubbard term *V* [70,71,72]. Here, *U* refers to the effective Coulomb repulsion between two electrons on the same BEDT-TTF molecule, while *V* refers to the effective Coulomb repulsion between two electrons on the neighboring BEDT-TTF molecules. The DFT calculations by Alemany et al. [68], however, suggest that the correlation effects within the organic BEDT-TTF layers are not sufficient for charge ordering in α-(BEDT-TTF)${\;\;}_{2}$I${\;\;}_{3}$, but that the structural changes within the inorganic I${\;\;}_{3}$ layers also need to be taken into account.

As mentioned in Section 1, the insulating CO state implies the existence of a correlation-induced CO gap $2\Delta_{\mathrm{CO}}$ at $E_{F}$. Indeed, the two most powerful probes for the detection of an energy gap, resistivity and infrared spectroscopy, confirmed a sudden opening of an energy gap at the CO transition temperature of $T_{\mathrm{CO}}=135$ K [66]. The opening of an energy gap was also inferred from the magnetic susceptibility measurements [73]. Our detailed transport study, however, showed that the resistivity in the CO phase does not follow a simple activated behavior, $\rho \left(T\right)\propto \exp\left(\Delta_{\mathrm{CO}}/T\right)$, expected from the opening of the CO gap 2Δ [69]. A detailed analysis based on the special logarithmic resistivity derivative X=−dlnρ/(dlnT) clearly indicated that the resistivity in the CO phase does not follow any variant of the VRH mechanism $\rho \left(T\right)\propto \exp{\left(T_{0}/T\right)}^{1/(d+1)}$ either, including 1D VRH (d=1), 2D VRH (d=2), and 3D VRH (d=3) [69].

**Figure 5:**
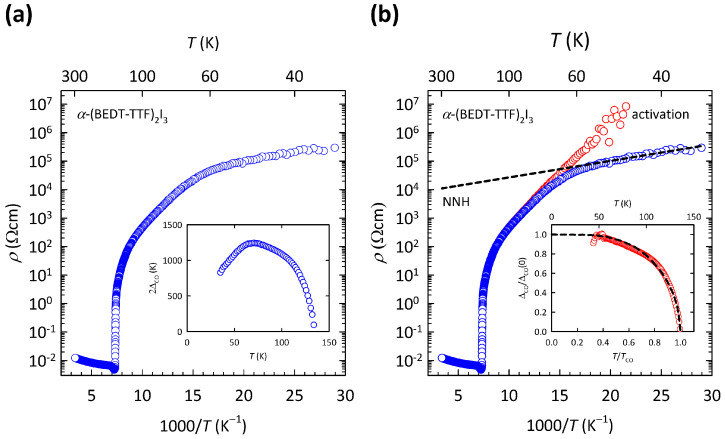
(**a**) Temperature dependence of resistivity for α-(BEDT-TTF)${\;\;}_{2}$I${\;\;}_{3}$ on a logρ-1/T plot. Inset shows the *T*-evolution of the corresponding energy gap $2\Delta_{\mathrm{CO}}$ extracted from a more general expression for activated behavior $\rho \left(T\right)=\rho_{0}\exp\left(\Delta_{\mathrm{CO}}\left(T\right)/T\right)$ (see text). (**b**) Decomposition of the measured resistivity for α-(BEDT-TTF)${\;\;}_{2}$I${\;\;}_{3}$ (blue symbols) into two parallel conductivity channels (see text): the NNH channel (black dashed line) and the activation channel (red symbols). The NNH channel is obtained by fitting the low-*T* data to the expression $\rho \left(T\right)\propto \exp\left(\Delta_{\mathrm{NNH}}/T\right)$. The activation channel is obtained by subtracting the NNH fit ($\rho_{\mathrm{NNH}}$) from the measured data ($\rho_{\mathrm{measured}}$) by using expression $1/\rho_{\mathrm{activation}}=1/\rho_{\mathrm{measured}}-1/\rho_{\mathrm{NNH}}$ (see text). Inset shows *T*-evolution of normalized energy gap $2\Delta_{\mathrm{CO}}$ extracted from the activation channel by using expression $\rho \left(T\right)=\rho_{0}\exp\left(\Delta_{\mathrm{CO}}\left(T\right)/T\right)$ (see text). The black dashed line in inset represents the normalized temperature dependence of the mean-field theoretical order parameter [74]. The figure is based on data from Ref. [69].

The results of our previous study [69] are illustrated here in Figure 5. As we can see in the logρ-1/T plot in Figure 5a, the resistivity of α-(BEDT-TTF)${\;\;}_{2}$I${\;\;}_{3}$ exhibits a sudden jump at $T_{\mathrm{CO}}$, and at low *T* it shows a complex behavior with a knee-like feature at T≈60 K. As we argued in Ref. [69], such *T* dependence might emerge if the CO gap $2\Delta_{\mathrm{CO}}$ changes with temperature, i.e., if the resistivity follows more general activated behavior $\rho \left(T\right)=\rho_{0}\exp\left(\Delta_{\mathrm{CO}}\left(T\right)/T\right)$. If a constant $\rho_{0}$ is set in such a way that the gap $2\Delta_{\mathrm{CO}}\left(T\right)$ vanishes at $T_{\mathrm{CO}}$, the *T* evolution of the gap can be extracted from the measured ρ(T) data. Such determined $2\Delta_{\mathrm{CO}}\left(T\right)$ is depicted here in the inset of Figure 5a. As can be seen, the extracted CO gap $2\Delta_{\mathrm{CO}}\left(T\right)$ first increases on cooling with decreasing *T*, then reaches a maximum value at T≈60 K and after that decreases with decreasing *T*, which at first sight seems unphysical. We note that the maximum value in $2\Delta_{\mathrm{CO}}\left(T\right)$ coincides with the knee-like feature in ρ(T).

A very similar temperature evolution of the energy gap was found in the organic conductors based on the tetramethyltetrathiafulvalene (TMTTF) molecule. The decrease in the energy gap at low *T* was there ascribed to the presence of some form of hopping conductivity [75]. Seeing that all forms of VRH are ruled out, the decrease in the CO gap at low *T* in the case of α-(BEDT-TTF)${\;\;}_{2}$I${\;\;}_{3}$ was ascribed to the NNH mechanism [69], which has the same *T* dependence as the simple activated behavior $\rho \left(T\right)\propto \exp\left(\Delta_{\mathrm{NNH}}/T\right)$. The presence of NNH in α-(BEDT-TTF)${\;\;}_{2}$I${\;\;}_{3}$ was additionally supported by our magnetoresistance measurements [69].

As we showed in Ref. [69], after subtracting the NNH fit ($\rho_{\mathrm{NNH}}$) from the measured resistivity data ($\rho_{\mathrm{measured}}$) in a parallel conductivity channel picture, the remaining resistivity $1/\rho_{\mathrm{remaining}}=1/\rho_{\mathrm{measured}}-1/\rho_{\mathrm{NNH}}$ now produces the CO gap $2\Delta_{\mathrm{CO}}\left(T\right)$ that almost perfectly follows the mean-field-like behavior. Such a procedure is shown here in Figure 5b. As we can see, by removing the hopping channel from the measured resistivity, we also removed the peculiar decrease of the CO gap below ≈60 K (compare insets of Figure 5a,b). In this way, the complex charge transport in the CO state of α-(BEDT-TTF)${\;\;}_{2}$I${\;\;}_{3}$ is separated into two simple contributions: the NNH channel that follows the exponential *T* dependence $\rho \left(T\right)\propto \exp\left(\Delta_{\mathrm{NNH}}/T\right)$ and the activation channel that follows the activated behavior with a mean-field like CO gap $\rho \left(T\right)\propto \exp(\Delta_{\mathrm{CO}}\left(T\right)/T)$. Close to the CO transition, the charge transport is dominated by the mean-field activation channel, while at the lowest *T*, it is dominated by the NNH channel. In the *T* region in between, the two channels compete, giving rise to the aforementioned knee-like feature at T≈60 K.

The presence of the NNH conductivity channel in α-(BEDT-TTF)${\;\;}_{2}$I${\;\;}_{3}$ implies a significant level of disorder within the conducting BEDT-TTF layers, which is unexpected, since the corresponding single crystals are nominally clean. However, early X-ray diffraction measurements detected intense diffuse lines already at room temperature, which were assigned to disorder within the I${\;\;}_{3}^{-}$ anion chains in the insulating layers [76]. We proposed, in Ref. [69], that this disorder within the insulating I${\;\;}_{3}$ layers is effectively transferred to the conducting BEDT-TTF layers via strong hydrogen bonds between the ethylene groups of the BEDT-TTF molecules and disordered I${\;\;}_{3}^{-}$ anion chains. The I${\;\;}_{3}^{-}$ disorder might also be responsible for the unusual properties found in infrared [66] and dielectric [69] spectroscopy data.

The presence of the NNH conductivity channel in α-(BEDT-TTF)${\;\;}_{2}$I${\;\;}_{3}$ further implies the existence of disorder-induced localized states at $E_{F}$, which is closer to the Anderson than to the CO insulators. On the other hand, the presence of the additional mean-field activation channel implies the existence of a well-defined energy gap at $E_{F}$, which is closer to the CO than the Anderson insulators. Counter-intuitively, the presence of disorder significantly decreases resistivity, as clearly visible in Figure 5b, since if there was no disorder, only the mean-field activation channel would be present and the resistivity would have much higher values at low-*T*. This counter-intuitive disorder-induced increase in conductivity resembles the behavior found in the κ-BEDT-TTF family, described in Section 2. In contrast to the κ-BEDT-TTF family, however, in which the charge transport is dominated by hopping among disorder-induced localized states at $E_{F}$, the charge transport in α-(BEDT-TTF)${\;\;}_{2}$I${\;\;}_{3}$ is dominated by activation across the correlation-induced energy gap, except at the lowest *T* where hopping conductivity takes over. Such behavior possibly indicates that the charge transport in α-(BEDT-TTF)${\;\;}_{2}$I${\;\;}_{3}$ is much less influenced by disorder than the charge transport in κ-BEDT-TTF family. The simultaneous presence of this correlation-induced gap, the disorder-driven hopping conductivity, and the counter-intuitive disorder-induced increase in conductivity in α-(BEDT-TTF)${\;\;}_{2}$I${\;\;}_{3}$ is discussed in Section 5.

## 4. La${\;\;}_{0.5}$Ca${\;\;}_{0.5}$MnO${\;\;}_{3}$

As a third example of a material in which the charge transport is affected by correlations and disorder, we consider the manganite La${\;\;}_{1-x}$Ca${\;\;}_{x}$MnO${\;\;}_{3}$ with x=0.5. This material belongs to a large family of manganese oxides $R_{1-x}A_{x}$MnO${\;\;}_{3}$, that became famous due to the colossal magnetoresistance (CMR) effect [77,78]. Here, *R* stands for a trivalent rare earth element or bismuth and *A* for a divalent alkaline earth element or lead. Except CMR, manganites are also interesting because of their very rich phase diagrams that contain MI transitions, charge ordering phenomena, long-range magnetic orders, and phase separation [4,79,80,81].

**Figure 6:**
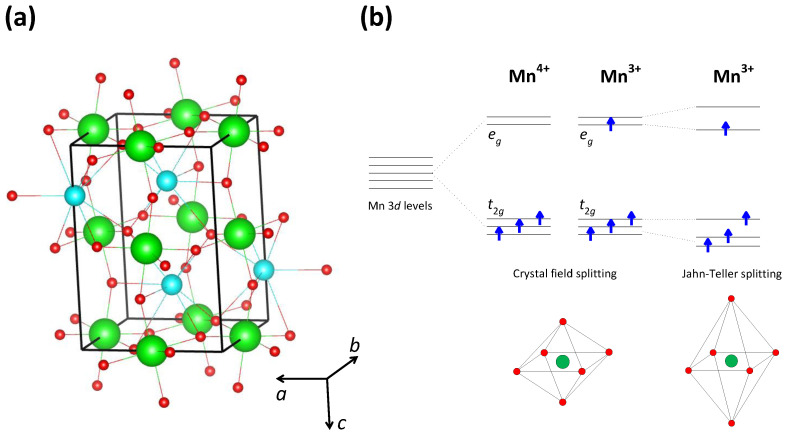
(**a**) Perovskite crystal structure of the parent compound LaMnO${\;\;}_{3}$. The unit cell is indicated by black lines. Lanthanum, manganese, and oxygen atoms are shown in cyan, green, and red, respectively. This figure is based on data from Ref. [84]. (**b**) Splitting of the Mn 3D levels into $e_{g}$ and $t_{2g}$ orbitals by the crystal field created by octahedral oxygen surrounding. In the case of Mn${\;\;}^{3+}$, there is an additional splitting due to the Jahn–Teller distortion of MnO${\;\;}_{6}$ octahedra (see text).

According to simple stoichiometry, La${\;\;}_{0.5}$Ca${\;\;}_{0.5}$MnO${\;\;}_{3}$ contains La${\;\;}^{3+}$, Ca${\;\;}^{2+}$, and O${\;\;}^{2-}$ ions, which means that Mn is in the so-called mixed valence state Mn${\;\;}^{3+}$/Mn${\;\;}^{4+}$. The electronic properties are expected to be mainly governed by Mn${\;\;}^{3+}$ and Mn${\;\;}^{4+}$, the only ions with open shells, as suggested also by studies based on density functional theory (DFT) [82,83]. Due to the perovskite crystal structure (Figure 6a), each Mn atom is octahedrally surrounded by six O atoms, so that there is the standard crystal-field splitting of the five Mn 3*d* orbitals into two high-energy $e_{g}$ orbitals and three low-energy $t_{2g}$ orbitals (Figure 6b). Because of the strong Hund coupling, each of these orbitals can host only one electron and therefore the three electrons of Mn${\;\;}^{4+}$ occupy the three low-energy $t_{2g}$ orbitals, while the four electrons of Mn${\;\;}^{3+}$ occupy the three low-energy $t_{2g}$ orbitals and one high-energy $e_{g}$ orbital. Due to the Hund coupling, the spins of all these electrons are oriented parallel to each other, so that Mn${\;\;}^{3+}$ and Mn${\;\;}^{4+}$ ions carry a magnetic dipole moment (see Figure 6b).

By taking into account the crystal-field splitting and the Hund coupling, we can therefore say that the Mn${\;\;}^{4+}$ and Mn${\;\;}^{3+}$ ions together produce six $t_{2g}$ orbitals and six $t_{2g}$ electrons, so according to the simple band theory, the band formed by these $t_{2g}$ orbitals is completely filled. On the other hand, the Mn${\;\;}^{4+}$ and Mn${\;\;}^{3+}$ ions together produce four $e_{g}$ orbitals, but only one $e_{g}$ electron, so the band formed by these $e_{g}$ orbitals is quarter-filled. Here, it is worth mentioning that such counting relies on the fact that in the La${\;\;}_{0.5}$Ca${\;\;}_{0.5}$MnO${\;\;}_{3}$ sample, Mn${\;\;}^{3+}$ and Mn${\;\;}^{4+}$ are present in the ratio of 1:1; more complicated counting is necessary for different ratios of Mn${\;\;}^{3+}$ and Mn${\;\;}^{4+}$ ions.

According to the simple band theory, therefore, valence $e_{g}$ band in La${\;\;}_{0.5}$Ca${\;\;}_{0.5}$MnO${\;\;}_{3}$ is quarter-filled, which implies that its nature is metallic and that all Mn ions carry equal charge, i.e., are present as Mn${\;\;}^{3.5+}$. Due to the aforementioned Hund coupling between $e_{g}$ and $t_{2g}$ electrons, the formation of the $e_{g}$-band strongly depends on the arrangement of magnetic dipole moments created by $t_{2g}$ electrons. Indeed, as shown by Zener back in 1950 [85], and later by Anderson et al. [86] as well as Kubo and Ohata [87], the effective transfer integral for the hop of an $e_{g}$ electron between Mn ions may be written as $t=t_{\max}\cos\left(\theta /2\right)$, where $t_{\max}$ is the maximal value of the transfer integral and θ is the angle between the magnetic moments of the Mn ions involved in the hopping process. This is the famous double-exchange model, according to which the maximum hopping probability occurs for parallel orientation of Mn magnetic moments (θ=0). In other words, the double-exchange model predicts that the formation of the $e_{g}$ band in manganites results in the metallic behavior accompanied by ferromagnetism. Such a ferromagnetic (FM) metallic ground state is indeed realized in La${\;\;}_{1-x}$Ca${\;\;}_{x}$MnO${\;\;}_{3}$, but only in the part of the phase diagram with x<0.5.

In the part of the phase diagram with x≥0.5, which includes the manganite La${\;\;}_{0.5}$Ca${\;\;}_{0.5}$MnO${\;\;}_{3}$ considered here, however, the experiments indicate that the ground state is in fact insulating [4,79,80,81,88]. This insulating behavior is most often ascribed to the CO localization of conducting charge carriers, in case of La${\;\;}_{0.5}$Ca${\;\;}_{0.5}$MnO${\;\;}_{3}$ within a quarter-filled band. Consequently, the theoretical models are usually based on a quarter-filled extended Hubbard Hamiltonian, where the most important is the extended Hubbard term *V*, which, in the case of manganites, refers to the effective Coulomb repulsion between two $e_{g}$ electrons on neighboring Mn ions. Due to this Coulomb repulsion, conduction $e_{g}$ electrons in La${\;\;}_{0.5}$Ca${\;\;}_{0.5}$MnO${\;\;}_{3}$ localize on every second Mn ion forming a charge-ordered state with a periodic array of Mn${\;\;}^{3+}$ and Mn${\;\;}^{4+}$ ions. More complicated CO structures are realized for other Ca-dopings *x*.

The ground state of manganites La${\;\;}_{1-x}$Ca${\;\;}_{x}$MnO${\;\;}_{3}$ with x≥0.5 is therefore usually described as a CO insulating phase [4,79,80,81]. In contrast to the metallic phase for x<0.5 which is accompanied by ferromagnetism, the CO insulating phase for x≥0.5 is accompanied by antiferromagnetism, as a consequence of the standard antiferromagnetic (AFM) superexchange interaction that occurs between the $t_{2g}$ electrons on neighboring Mn ions. This long-range AFM order reinforces the insulating nature of the CO phase by an additional localization of conduction $e_{g}$ electrons through the aforementioned double-exchange mechanism. Namely, according to the double-exchange mechanism, the hopping of $e_{g}$ electrons is given by $t=t_{\max}\cos\left(\theta /2\right)$ so that the delocalization vanishes when $\theta =180^{{}^{\circ}}$, i.e., for the AFM alignment of Mn magnetic moments.

The insulating nature of the CO phase is also reinforced by an additional localization of conduction $e_{g}$ electrons through the Jahn–Teller effect. Namely, in contrast to the Mn${\;\;}^{4+}$ ion, the Mn${\;\;}^{3+}$ ion is Jahn–Teller-active, so in the case of Mn${\;\;}^{3+}$, there is an additional splitting of $e_{g}$ (and $t_{2g}$) orbitals caused by the deformation of an Mn${\;\;}^{3+}$O${\;\;}_{6}$ octahedron (see Figure 6b). This means that an $e_{g}$ electron can hop only from an Mn${\;\;}^{3+}$ to an Mn${\;\;}^{4+}$ ion and that each such hop is accompanied by a structural change in the MnO${\;\;}_{6}$ octahedra. Such behavior leads to strong electron–phonon coupling effects that hinder the free movement of conduction $e_{g}$ electrons. Indeed, an insulator-like polaronic conductivity has been observed in manganites at high temperatures [89,90,91,92,93,94], and the Jahn–Teller effect has been suggested to play an essential role in the stabilization of the CO phase [95].

As mentioned in Section 1, the CO insulating phase implies the existence of a correlation-induced CO gap $2\Delta_{\mathrm{CO}}$ at $E_{F}$. However, our resistivity measurements on La${\;\;}_{0.5}$Ca${\;\;}_{0.5}$MnO${\;\;}_{3}$ ceramic samples clearly indicated the absence of the simple activated behavior $\rho \left(T\right)\propto \exp\left(\Delta_{\mathrm{CO}}/T\right)$ down to the lowest accessible temperatures [96]. Our detailed analysis of the resistivity, in fact, showed that charge transport in La${\;\;}_{0.5}$Ca${\;\;}_{0.5}$MnO${\;\;}_{3}$ at low *T* takes place via the VRH mechanism $\rho \left(T\right)\propto \exp{\left(T_{0}/T\right)}^{1/(d+1)}$. The best fit was obtained for VRH in three dimensions (d=3), in accordance with the perovskite crystal structure.

**Figure 7:**
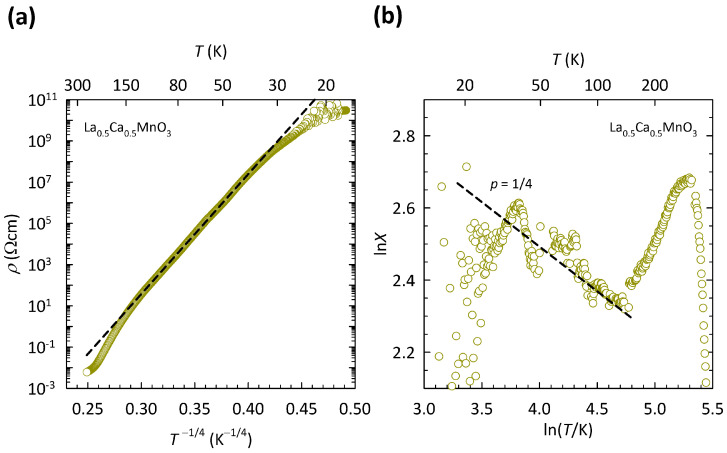
(**a**) Temperature dependence of resistivity for La${\;\;}_{0.5}$Ca${\;\;}_{0.5}$MnO${\;\;}_{3}$ ceramics on a logρ-$T^{-1/4}$ plot. Black dashed line is a fit to the 3D VRH mechanism, $\rho \left(T\right)\propto \exp{\left(T_{0}/T\right)}^{1/4}$. (**b**) *T* dependence of the corresponding special logarithmic resistivity derivative $X=-d\ln\rho /\left(d\ln T\right)=p{\left(C/T\right)}^{p}$ on a double logarithmic plot. Black dashed line corresponds to slope p=1/4 for 3D VRH, where *p* is the exponent in a general expression for resistivity $\rho \left(T\right)\propto \exp{\left(C/T\right)}^{p}$ (see text). The figure is based on data from Ref. [96].

The presence of the 3D VRH mechanism in La${\;\;}_{0.5}$Ca${\;\;}_{0.5}$MnO${\;\;}_{3}$ ceramics, indicated in our previous study [96], is depicted here in Figure 7a. The data are shown on a logρ-$T^{-1/4}$ plot, suitable for 3D VRH. As can be clearly seen, the ρ(T) data in the *T* range of ≈30−150 K can be fitted to the 3D VRH mechanism almost perfectly, which is indicated by a black dashed line. We note that the fit to 3D VRH covers the increase in resistivity by almost eight orders of magnitude and deviates only at the lowest *T* below ≈30 K, where the resistance of the sample becomes very large and therefore hard to measure, as also indicated by an increased scatter in the data. The deviation from 3D VRH at high *T* (>150 K) resembles the crossover to the NNH regime. However, as we argued in detail in Ref. [96], this deviation at high *T* is related to the phase transition from the CO ground state to a paramagnetic insulating state that in La${\;\;}_{0.5}$Ca${\;\;}_{0.5}$MnO${\;\;}_{3}$ takes place at $T_{\mathrm{CO}}\approx 200$ K and is discussed later.

The presence of 3D VRH in La${\;\;}_{0.5}$Ca${\;\;}_{0.5}$MnO${\;\;}_{3}$ was additionally confirmed in Ref. [96] by directly extracting exponent *p* in a general expression for resistivity $\rho \left(T\right)\propto \exp{\left(C/T\right)}^{p}$ with the use of special logarithmic resistivity derivative X=−dlnρ/(dlnT), as described in Section 2. Derivative *X* for La${\;\;}_{0.5}$Ca${\;\;}_{0.5}$MnO${\;\;}_{3}$ is shown here in Figure 7b. As we can see, the slope of the ln*X*-ln*T* curve in the *T* range of 30–150 K agrees very well with the value of p=1/4 expected for the 3D VRH mechanism. No crossover to value p=1 for NNH at high *T* has been found, in line with presence of the CO phase transition at $T_{\mathrm{CO}}\approx 200$ K [96].

The presence of the 3D VRH mechanism in La${\;\;}_{0.5}$Ca${\;\;}_{0.5}$MnO${\;\;}_{3}$ implies the existence of localized states at $E_{F}$ induced by a significant level of disorder, which is most plausibly ascribed to the La/Ca substitution. This means that the insulating behavior of La${\;\;}_{0.5}$Ca${\;\;}_{0.5}$MnO${\;\;}_{3}$ stems from localization of conduction $e_{g}$ electrons driven by disorder effects, rather than by correlation effects associated with charge ordering. In other words, La${\;\;}_{0.5}$Ca${\;\;}_{0.5}$MnO${\;\;}_{3}$ seems to be closer to the Anderson insulators that have a finite density of (localized) states at $E_{F}$, than to the CO insulators that have an energy gap at $E_{F}$.

The presence of Anderson-type localization of conduction $e_{g}$ electrons in La${\;\;}_{0.5}$Ca${\;\;}_{0.5}$MnO${\;\;}_{3}$ implies that the increase of disorder results in the more localized states at $E_{F}$ and therefore in stronger insulating behavior, i.e., steeper dρ/dT. An obvious way of tuning the level of structural disorder in La${\;\;}_{1-x}$Ca${\;\;}_{x}$MnO${\;\;}_{3}$ is by changing La/Ca substitution (*x*). Such substitution, however, besides a change in disorder level, also changes the chemical potential, i.e., the doping level. In order to avoid such problems, our group rather focused on the grain size, which is a very convenient way of tuning the level of disorder in manganite ceramic (polycrystalline) samples since it can be easily controlled by annealing temperature during the synthesis. Recently, we reported such a transport study on La${\;\;}_{0.5}$Ca${\;\;}_{0.5}$MnO${\;\;}_{3}$ ceramic samples with three different grain sizes: 4000 nm, 400 nm, and 40 nm [96].

**Figure 8:**
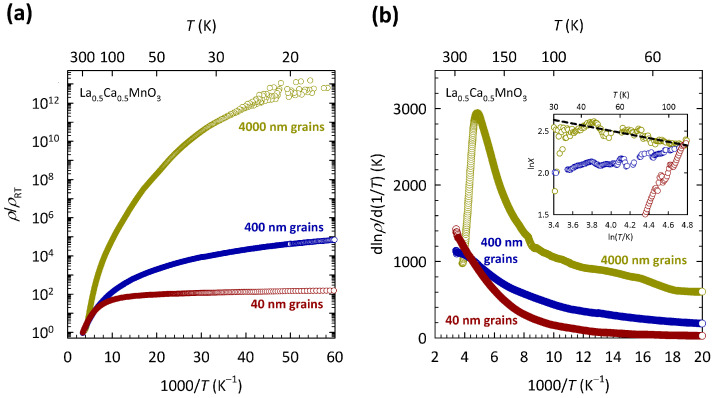
Temperature dependence of (**a**) resistivity normalized to room temperature value $\rho /\rho_{\mathrm{RT}}$ and (**b**) corresponding logarithmic resistivity derivative dlnρ/dln(1/T) for La${\;\;}_{0.5}$Ca${\;\;}_{0.5}$MnO${\;\;}_{3}$ ceramic samples with different grain sizes: 4000 nm (dark yellow symbols), 400 nm (dark blue symbols), and 40 nm (dark red symbols). The data for the sample with 4000 nm grains are the same as in Figure 7a. Maximum in dlnρ/dln(1/T) corresponds to the CO transition (see text). Inset shows the *T* dependence of special logarithmic resistivity derivative $X=-d\ln\rho /\left(d\ln T\right)=p{\left(C/T\right)}^{p}$ on a double logarithmic plot. Black dashed line corresponds to slope p=1/4 for 3D VRH, where *p* is the exponent in a general expression for resistivity $\rho \left(T\right)\propto \exp{\left(C/T\right)}^{p}$ (see text). The figure is based on data from Ref. [96].

The results of this study are shown in Figure 8. The resistivities in Figure 8a are normalized to room temperature values $\rho_{\mathrm{RT}}$ in order to highlight the difference in the *T* dependence between the samples with different grain sizes. The values of $\rho_{\mathrm{RT}}$ are approximately 6 mΩcm, 1.5 Ωcm, and 4.4 Ωcm for the samples with 4000, 400, and 40 nm grains, respectively. The data for the sample with 4000 nm grains are the same as in Figure 7. As we can see, the reduction in the grain size by two orders of magnitude leads to an enormous decrease in $\rho /\rho_{\mathrm{RT}}$, at the lowest *T* by more than ten orders of magnitude. This suggests that a large increase in the level of structural disorder in La${\;\;}_{0.5}$Ca${\;\;}_{0.5}$MnO${\;\;}_{3}$, induced by grains, pushes the system towards a metallic state, instead of promoting more insulating behavior. Such behavior is counter-intuitive and is in sharp contrast with the simple Anderson localization scenario proposed earlier. Here, it is worth mentioning that similar grain-size effects have been observed in other members of the manganite family as well [97,98], possibly pointing towards a common feature of charge transport in manganites.

The behavior of charge transport in La${\;\;}_{0.5}$Ca${\;\;}_{0.5}$MnO${\;\;}_{3}$ is therefore not fully consistent with either the Mott or the Anderson localization scenario and thus resembles the behavior found in organic conductor κ-(BEDT-TTF)${\;\;}_{2}$Cu${\;\;}_{2}$(CN)${\;\;}_{3}$ and its sister compounds described in Section 2, as well as in organic conductor α-(BEDT-TTF)${\;\;}_{2}$I${\;\;}_{3}$ described in Section 3. In the case of La${\;\;}_{0.5}$Ca${\;\;}_{0.5}$MnO${\;\;}_{3}$, the disorder-induced drop in resistivity is so large that the 3D VRH mechanism is completely lost already in the sample with medium grains (400 nm), while the sample with the smallest grains (40 nm) almost exhibits a true metallic behavior (dρ/dT>0). As we showed in Ref. [96], the disappearance of 3D VRH coincides with the disappearance of the CO transition, which is illustrated here in Figure 8b. As we can see, the La${\;\;}_{0.5}$Ca${\;\;}_{0.5}$MnO${\;\;}_{3}$ ceramic sample with the largest grains (4000 nm) shows a clear maximum in dlnρ/dln(1/T) at ≈200 K. Such a maximum in dlnρ/dln(1/T) is typical for La${\;\;}_{1-x}$Ca${\;\;}_{x}$MnO${\;\;}_{3}$ compounds in the CO/AFM insulating part of phase diagram x≥0.5, and as shown in Refs. [99,100], its position agrees well with the temperature at which the CO transition is expected to occur according to the phase diagram. The maximum in dlnρ/dln(1/T) at ≈200 K in Figure 8b shows a fair agreement with the phase diagram as well.

As we can see, the fingerprint of the CO transition (maximum in dlnρ/dln(1/T)) is largely suppressed with reducing the grain size and is no longer present in the samples with medium (400 nm) and smallest grains (40 nm). The disappearance of 3D VRH is indicated in the inset of Figure 8b, which clearly shows that slope *p* in the ln*X*-ln*T* plot for the samples with 400 nm and 40 nm grains not only deviates from the value of p=1/4 expected for 3D VRH, but even becomes negative. Such simultaneous collapse of the 3D VRH mechanism and the CO phase indicates that the localization of conduction $e_{g}$ electrons in La${\;\;}_{0.5}$Ca${\;\;}_{0.5}$MnO${\;\;}_{3}$ is driven by combined effects of the correlations and disorder potential. The ways in which these joint effects explain the counter-intuitive approach to the metallic state in La${\;\;}_{0.5}$Ca${\;\;}_{0.5}$MnO${\;\;}_{3}$ with the increase in disorder is discussed in the next section.

## 5. Discussion

The overview of our long-standing study on the selected organic conductors and manganites presented in Section 2, Section 3 and Section 4 revealed striking similarities between all these materials. While the conventional band theory predicts a metallic state, advanced theoretical considerations suggest the correlation-induced (Mott or CO) insulating phase to be more relevant. Our overall electrical resistivity data confirm the insulator-like behavior but, surprisingly, instead of the expected simple activation, uncover the VRH and/or NNH conduction mechanism, thus indicating the disorder-induced (Anderson-like) insulating phase. In sharp contrast to the Anderson scenario, however, the increase in disorder, instead of driving more insulating behavior, counter-intuitively pushes the system towards the metallic state. Therefore, the selected materials show a common pattern of behavior in the charge sector featuring both correlation-induced (Mott/CO) and disorder-induced (Anderson) localization, meaning that neither of the two mechanisms can fully capture the behavior of their charge transport.

**Figure 9:**
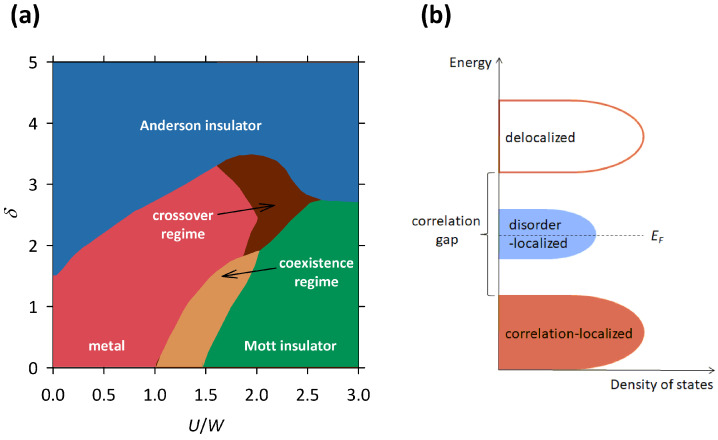
(**a**) An illustration of the Mott–Anderson phase diagram (after Ref. [48]), where the correlation strength is denoted by U/W and the disorder strength is denoted by δ (see text). (**b**) A simplified illustration of the profile of density of states around $E_{F}$ for a Mott–Anderson insulator in the part of the phase diagram with intermediate correlation strength U/W and small disorder strength δ. There is a clear distinction between correlation-induced localized states, which are separated by the correlation gap at $E_{F}$ from the conduction band, and disorder-induced localized states that reside in vicinity of $E_{F}$.

During the last twenty years, several theoretical groups in the world have developed more complex theories of electron localization that simultaneously take into account both Mott and Anderson types of localization mechanisms. These theories [44,48,49] study the electron localization in the presence of correlations as well as disorder, which results in a complicated phase diagram shown in Figure 9a. The *x*-axis in this phase diagram corresponds to the correlation strength U/W, and the *y*-axis corresponds to disorder strength δ.

As we can see, when disorder strength δ is small, an increase in correlation strength U/W leads to a transition from the metal to the Mott insulator, which corresponds to the standard scenario of the Mott localization. In this case, the theory predicts that the correlations open a well-defined energy gap at $E_{F}$ as the system crosses the MI transition. In the vicinity of the MI transition, there is a narrow regime where the metallic and Mott insulating phases coexist [48]. On the other hand, when U/W is small, an increase in δ leads to a transition from the metal to the Anderson insulator, which corresponds to the standard scenario of the Anderson localization. In this case, the theory predicts that the disorder changes the character of the states at $E_{F}$ from delocalized to localized as the system crosses the MI transition, without opening of an energy gap. When both U/W and δ are large, the theory predicts a direct transition between the Mott and the Anderson insulator, while for intermediate values of U/W and δ, there is a narrow crossover regime in between [48]. In both these cases, the localization effects caused by the correlations and the disorder reinforce each other, leading to stronger insulating behavior.

The most interesting are the cases in which one parameter is small while the other takes an intermediate value, since then the localization effects caused by the correlations and the disorder drive the system in opposite directions. For example, when U/W is small and δ takes an intermediate value, increase in U/W, instead of reinforcing the insulating behavior, counter-intuitively pushes the system from Anderson insulating towards the metallic phase (see Figure 9a). Such behavior is within the theory of Mott–Anderson localization ascribed to the screening of the disorder potential that becomes stronger with the increase in U/W [48].

For us, the most important is the case when δ is small and U/W takes an intermediate value, since here an increase in δ, instead of reinforcing the insulating behavior, counter-intuitively pushes the system from the Mott insulating towards the metallic phase. This is exactly what we observed in the selected organic conductors and manganites presented in Section 2, Section 3 and Section 4. Such behavior is within the Mott–Anderson localization theory ascribed to the presence of a well-defined correlation gap at $E_{F}$, which with increasing disorder becomes progressively filled in with localized states at $E_{F}$, thus making the system more conductive [48] (see Figure 9b).

The presence of these theoretically predicted localized states at $E_{F}$ perfectly explains the experimentally observed hopping conductivity in all presented materials: 2D VRH and NNH in κ-(BEDT-TTF)${\;\;}_{2}$Cu${\;\;}_{2}$(CN)${\;\;}_{3}$ (Figure 2) and its sister compounds κ-(BEDT-TTF)${\;\;}_{2}$Ag${\;\;}_{2}$(CN)${\;\;}_{3}$ and κ-(BEDT-TTF)${\;\;}_{2}$B(CN)${\;\;}_{4}$ (Figure 3), 3D VRH in La${\;\;}_{0.5}$Ca${\;\;}_{0.5}$MnO${\;\;}_{3}$ (Figure 7), and NNH in α-(BEDT-TTF)${\;\;}_{2}$I${\;\;}_{3}$ (Figure 5). On the other hand, the presence of the theoretically predicted correlation gap at $E_{F}$ perfectly explains the experimentally observed maximum in the logarithmic resistivity derivative dlnρ/dln(1/T) in La${\;\;}_{0.5}$Ca${\;\;}_{0.5}$MnO${\;\;}_{3}$ (Figure 8b) and the conductivity channel with a mean-field activation in α-(BEDT-TTF)${\;\;}_{2}$I${\;\;}_{3}$ (Figure 5) that are both associated with the development of the CO phase.

Most importantly, the theoretically predicted simultaneous presence of the localized states and the correlation gap at $E_{F}$ perfectly explains the experimentally observed counter-intuitive disorder-induced increase in conductivity in all presented materials. As the disorder grows, so does the density of localized states at $E_{F}$ and consequently the conductivity of the system. Besides conductivity, increase in the density of states at $E_{F}$ also affects the Hall effect. Indeed, in the case of κ-(BEDT-TTF)${\;\;}_{2}$Cu${\;\;}_{2}$(CN)${\;\;}_{3}$, κ-(BEDT-TTF)${\;\;}_{2}$Ag${\;\;}_{2}$(CN)${\;\;}_{3}$, and κ-(BEDT-TTF)${\;\;}_{2}$B(CN)${\;\;}_{4}$, we observed a large difference in their Hall carrier density. The most ordered κ-(BEDT-TTF)${\;\;}_{2}$B(CN)${\;\;}_{4}$ with the smallest conductivity was found to also have the smallest Hall carrier density, while the most disordered κ-(BEDT-TTF)${\;\;}_{2}$Cu${\;\;}_{2}$(CN)${\;\;}_{3}$ with the largest conductivity was found to also have the largest Hall carrier density [37].

The theoretically predicted simultaneous presence of the localized states and the correlation gap at $E_{F}$ also perfectly explains the experimentally observed parallel conductivity channels in α-(BEDT-TTF)${\;\;}_{2}$I${\;\;}_{3}$ (Figure 5b). Here, the NNH channel refers to the charge carriers that hop among disorder-induced localized states at $E_{F}$, while the mean-field activation channel refers to the charge carriers that are thermally excited across the correlation-induced (CO) gap (Figure 9b). Such two parallel conductivity channels might also explain the behavior of charge transport in κ-(BEDT-TTF)${\;\;}_{2}$Ag${\;\;}_{2}$(CN)${\;\;}_{3}$ and κ-(BEDT-TTF)${\;\;}_{2}$B(CN)${\;\;}_{4}$ at the highest *T*, where the exponent *p* from general expression for resistivity $\rho \left(T\right)\propto \exp{\left(C/T\right)}^{p}$ exhibits complex *T* dependence that is consistent with neither the simple activation nor any form of VRH.

Here, it is worth mentioning that such behavior resembles the behavior of amorphous semiconductors [15]. When prepared in the crystalline form, these materials are band gap insulators, meaning that they have a well-defined energy gap at $E_{F}$ between the valence and the conduction band. When prepared in the amorphous form, the valence and the conduction bands are still present, but the energy gap acquires additional structure, the most important aspect of which is the appearance of localized states at $E_{F}$. The conductivity of the amorphous form is higher compared to the crystalline form and charge transport at low temperatures usually takes place via some form of hopping conductivity. At higher temperatures, there could be an additional parallel conductivity channel that involves activation of charge carriers across the energy gap. Therefore, despite the fact that the disorder level is as high as it can be, the band gap is still there, and the amorphous semiconductors preserve the insulating nature.

In contrast to such band gaps in amorphous semiconductors, which are opened by the structure, the gaps opened by correlations are much less stable, and for sufficiently high disorder levels, they collapse, giving rise to insulator-to-metal transitions. Such a scenario might be relevant for La${\;\;}_{0.5}$Ca${\;\;}_{0.5}$MnO${\;\;}_{3}$ ceramic samples and could explain the experimentally observed simultaneous disappearance of the 3D VRH mechanism and CO transition (Figure 8b). Namely, it could be that the disorder level in the ceramic samples with 400 nm and 40 nm grains is sufficiently high to at least partially close the CO gap and therefore bring delocalized states close to $E_{F}$. Indeed, the sample with 40 nm grains exhibits almost a true metallic conduction at low *T*. As we showed in Ref. [96], the approach to the metallic state is accompanied by a large increase in ferromagnetic fingerprints, in line with the double-exchange mechanism that strongly couples the charge transport of $e_{g}$ electrons and the magnetic structure of $t_{2g}$ electrons. Considering that similar grain-size effects have also been observed in other manganite compounds [97,98], these conclusions appear to be generally relevant for manganites.

In order to fully close the CO gap and induce the ferromagnetic metallic state in La${\;\;}_{0.5}$Ca${\;\;}_{0.5}$MnO${\;\;}_{3}$, the disorder level needs to be further increased, which means that the grain size has to be tuned to even smaller values. Such a procedure is possible in principle, but very demanding experimentally. Even more demanding is the case of the selected organic conductors, in which in order to fully close the correlation gap and induce the metallic state, the level of disorder needs to be tuned sufficiently high by means of chemical substitution. The same applies also in the opposite limit. Namely, in all selected materials, it is very hard to tune the level of disorder sufficiently low for there to be a pure correlation gap without in-gap localized states. In fact, as can be clearly seen in Figure 3a and Figure 8a, the level of disorder can be easily tuned only in a limited range and in large discrete steps, which prevents us from studying fine changes in charge transport across insulator-to-metal transitions in selected materials.

Such fine changes across the insulator-to-metal transitions in the selected materials could be studied by varying the correlation strength instead, which in the case of the organic conductors is easily tuned by hydrostatic pressure and in the case of manganites by magnetic field. A recent study on κ-(BEDT-TTF)${\;\;}_{2}$Cu${\;\;}_{2}$(CN)${\;\;}_{3}$ under pressure indicated that the Mott insulator-to-metal transition has a percolative nature and therefore is not accompanied by simple closing of the correlation gap, but with a spatial coexistence of correlated metallic and insulating regions [101]. A similar percolative nature of the insulator-to-metal transition has also been reported in α-(BEDT-TTF)${\;\;}_{2}$I${\;\;}_{3}$ [102] and manganites La${\;\;}_{0.5}$Ca${\;\;}_{0.5}$Mn${\;\;}_{1-x}$Al${\;\;}_{x}$O${\;\;}_{3-\delta }$ [103]. Here, the recent development of new computational approaches based on machine learning is of utmost importance in unraveling fine details of the metal–insulator transitions in strongly correlated systems [104].

In summary, in the present review, we showed that in contrast to common views in the literature, many aspects of the transport data on the selected organic conductors from the BEDT-TTF family and manganite La${\;\;}_{0.5}$Ca${\;\;}_{0.5}$MnO${\;\;}_{3}$ ceramics are consistent with neither the pure Mott nor the pure Anderson localization scenario. In fact, we showed that the charge transport in the selected materials is strongly influenced by combined effects of correlations and disorder and therefore can be interpreted only within the much more advanced and very rarely considered theories of Mott–Anderson localization. Furthermore, we showed that the charge transport in all selected materials follows some form of the variable range hopping and/or nearest neighbor hopping. This is not a trivial finding, because it implies that the nature of these well-known mechanisms, typical for non-interacting electrons in disordered systems, is not changed in the presence of strong electron correlations. Very similar conclusions were inferred in our most recent study on selected mangnanite La${\;\;}_{1-x}$Ca${\;\;}_{x}$MnO${\;\;}_{3}$ thin films with 0.5≤x≤0.75 [105], and the same conclusions could be applied to the transport study by Köhler et al. on selected organic conductors from the TMTTF family [75] as well. All these findings together strongly suggest that Mott–Anderson localization might be a widely spread phenomenon within the strongly correlated materials and that therefore deserves a lot more attention from both the experimental and theoretical sides. Here, of utmost importance may be studies based on angle-resolved photoemission spectroscopy or optical conductivity, which could offer more insights into the profile of density of states around the Fermi level in these materials.

## 6. Conclusions

In this paper, we present an overview of our long-standing research on selected organic conductors and manganites that reveals a common pattern in the behavior of their charge transport. The most important aspects are the presence of hopping transport and a counter-intuitive increase in conductivity with increase in disorder. We argue that such behavior implies the localization of conducting charge carriers driven by the combined effects of correlations and disorder, which can be understood only within very few theories of Mott–Anderson localization. The basic idea is that the correlations open an energy gap at the Fermi level, thus promoting the insulating behavior, while the disorder fills in the gap with localized states at the Fermi level, thus increasing conductivity. We believe that such behavior, besides within the materials presented here, is to be found in other related materials as well, and therefore might be a common feature of strongly correlated systems. We sincerely hope that these findings will stimulate further experimental as well as theoretical research on the interplay between correlations and disorder, which is a very important but rarely studied phenomenon within condensed matter physics.

## Data Availability

All data presented in this article were already published in our previous studies.
